# Cardioprotection: A Review of Current Practice in Global Ischemia and Future Translational Perspective

**DOI:** 10.1155/2014/325725

**Published:** 2014-09-08

**Authors:** Andreas Habertheuer, Alfred Kocher, Günther Laufer, Martin Andreas, Wilson Y. Szeto, Peter Petzelbauer, Marek Ehrlich, Dominik Wiedemann

**Affiliations:** ^1^Department of Cardiac Surgery, Vienna General Hospital, Medical University of Vienna, Waehringer Guertel 18-20, 1090 Vienna, Austria; ^2^Division of Cardiovascular Surgery, University of Pennsylvania Medical Center, 3400 Spruce Street, Philadelphia, PA 19104, USA; ^3^Department of Dermatology, Vienna General Hospital, Medical University of Vienna, Waehringer Guertel 18-20, 1090 Vienna, Austria

## Abstract

The idea of protecting the heart from ischemic insult during heart surgery to allow elective cardiac arrest is as old as the idea of cardiac surgery itself. The current gold standard in clinical routine is a high potassium regimen added either to crystalloid or blood cardioplegic solutions inducing depolarized arrest. Ongoing patient demographic changes with increasingly older, comorbidly ill patients and increasing case complexity with increasingly structurally abnormal hearts as morphological correlate paired with evolutions in pediatric cardiac surgery allowing more complex procedures than ever before *redefine requirements for cardioprotection*. Many, in part adversarial, regimens to protect the myocardium from ischemic insults have entered clinical routine; however, functional recovery of the heart is still often impaired due to perfusion injury. Myocardial reperfusion damage is a key determinant of postoperative organ functional recovery, morbidity, and mortality in adult and pediatric patients. There is a discrepancy between what current protective strategies are capable of and what they are expected to do in a rapidly changing cardiac surgery community. An increased understanding of the molecular players of ischemia reperfusion injury offers potential seeds for new cardioprotective regimens and may further displace boundaries of what is technically feasible.

## 1. Introduction

For the majority of cardiac surgical interventions arresting the heart is inevitable, with systemic arterial perfusion and oxygenation being transferred to a heart lung machine. Until the present day, cardioplegic arrest remains the gold standard of cardioprotection and requires a potassium rich solution sending the heart into a depolarized arrest [1]. Despite its almost universal usage, cardioplegia in its current form is associated with potential downsides rendering those cardioprotective regimens a less than optimal choice in certain clinical situations and certain patient collectives.

25% of the population over 75 years suffers from symptoms of cardiovascular disease [2], and as the elderly represent the fastest growing population demographic in industrialized nations, the proportion of elderly patients being evaluated for cardiac surgery is only expected to increase (the average age of cardiac surgical patients increased from 55.8 years to 68.8 years in the course of the last decade [3]). In general, the elderly represent a comorbidly ill patient population with a higher perioperative risk. Factors influencing operative risk include age >70, female sex, renal impairment, extracardiac arteriopathy, chronic lung disease, pulmonary hypertension, insulin dependent diabetes, NYHA III/IV, and ejection fraction <50%. This is especially important in the present light of change in the field of interventional cardiology offering catheter-guided approaches to an increasingly larger patient cohort causing a shift in cardiac surgery away from isolated “simple” procedure towards more complex interventions [4], sometimes in the very old and the severely ill [1, 3]. This increase in case complexity in a changing patient population is especially relevant for patients with impaired ventricles associated with left ventricular hypertrophy (LVH) and heart failure where it is generally acknowledged that current methods of myocardial protection are inadequate. LVH increases myocardial workload and renders hypertrophic hearts more susceptible to ischemic injury [5] and impaired recovery postoperatively.

To develop new approaches towards innovative cardioprotective regimens it is increasingly important to understand the pathophysiologic and molecular players of ischemia as a two-fold phenomenon in which ischemic injury is only part of the truth and subsequent reperfusion injury has the potential to grossly outweigh the primary ischemic insult [6]. Myocardial reperfusion damage following cardioplegic ischemic arrest is a key determinant of postoperative organ functional recovery, morbidity, and mortality in adult and pediatric patients undergoing open-heart surgery and has the potential to cause protracted organ recovery, myocardial stunning, and acute myocardial infarction.

This paper reviews and compares current clinical regimens of cardioprotection via elective global ischemia induction and draws attention to potential avenues for innovative therapeutic approaches with the potential of translational application in future clinical trials, thereby highlighting management of ischemia reperfusion injury as a central dogma.

## 2. Electrophysiological Concepts: Induction of Arrest

**Figure 1:**
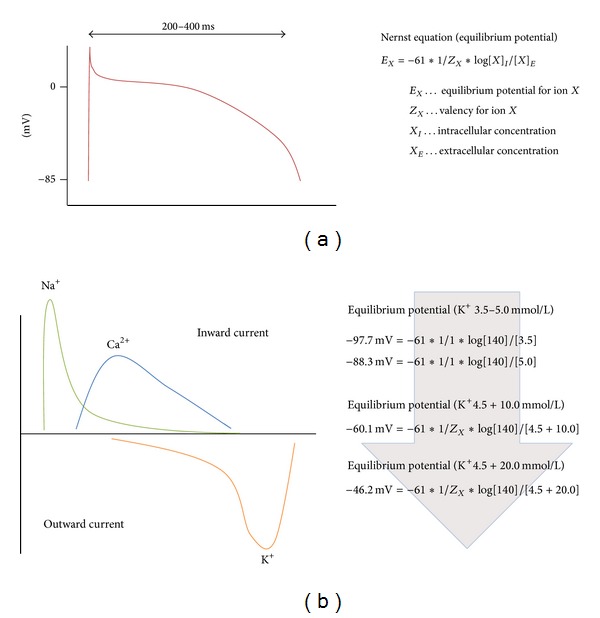
(a) Action potential for cardiac myocytes and (b) ion flux occurring during each cardiac myocyte action potential. Nernst equation on the right-hand side illustrating membrane potential changes upon modification of the extracellular K^+^ ion concentration.

Sending the heart into a diastolic flaccid arrest requires understanding of the underlying electrophysiology principles. Hyperkalemia (as the current clinical practice either via blood cardioplegia or crystalloid cardioplegic solution, Table 1) changes the cellular resting membrane potential (*E*_*m*_) of cardiac myocytes towards a less negative value (i.e., closer to zero). The resting membrane potential is largely maintained via an adenosine triphosphate (ATP) driven primary active 3Na^+^/2K^+^ exchange pump creating both chemical and electric gradients across the cellular membrane and via a passive K^+^ outward flux. As the cardiac myocyte membrane is most permeable to K^+^ ions but relatively impermeable to other ions, *E*_*m*_ potential is close to the K^+^ equilibrium potential of −91 mV (Nernst equation, Figure 1) and approaches −85 mV (Goldman-Hodgkin-Katz voltage equation).

The action potential (AP) is the result of an orchestrated activation of various voltage-gated channels located in the cell membrane (Figure 1). Upon stimulation by the sinuatrial node, voltage dependent Na^+^ channels open and allow rapid sodium ion influx and depolarization of the cardiac myocyte cell membrane to about +20 mV. The threshold potential for fast Na^+^ channel opening is about −65 mV. Na^+^ channels become inactivated within a split second and *E*_*m*_ would return to normal unless L-type Ca^2+^ channels open and allow further influx of positively charged ions, characteristically prolonging the AP in a plateau-like fashion. For most parts of the plateau phase Ca^2+^ influx and K^+^ outward directed back diffusion is balanced; however, as the membrane potential reaches more negative values, Ca^2+^ channels close and delayed K^+^ rectifiers send *E*_*m*_ back to normal and terminate yet another AP. Electromechanical coupling and force generation evolves as Ca^2+^ influx via dihydropyridine receptors of the myocyte cell membrane causes cytosolic Ca^2+^ release from the sarcoplasmic reticulum via ryanodine receptors (calcium induced calcium release). In a physiologic state with extracellular K^+^ levels of 3.5–5.0 mmol/L, *E*_*m*_ approaches −85 mV. One can easily calculate that an elevation of extracellular K^+^ towards 16.2 mmol/L decreases *E*_*m*_ to about −60 mV (Figure 1) well beyond the Na^+^ channel threshold, not allowing any further myocyte AP propagation generated by the sinuatrial node (SAN) because fast Na^+^ channels remain inactivated. As this state does not allow any repolarisation either the current clinical practice of hyperkalemic cardiolegia induction is called depolarized arrest. The institution of cardioplegic arrest ensures that myocardial oxygen consumption (MVO2) is significantly reduced, as is the ATP depletion characteristic of severe ischemia [7–9].

## 3. Cardioprotection: A Strategic Comparison of Current Clinical Practice

There is no doubt that adequate myocardial protection plays a key role in achieving successful outcomes in cardiac surgery. Many methods to achieve and maintain sustained electromechanical quiescence have been advocated and obviously few institutions share identical protocols which makes comparison in large multicenter studies and meta-analysis difficult. Nevertheless, we provide a comprehensive review of the most important clinical trials in Table 3. The spectrum of strategies has led to the creation of, in part, adversarial positions. In general, blood-based or crystalloid-based solutions are used as potassium-containing transport medium. Blood cardioplegia is mixed in a ratio of 1 : 4 (1 part of crystalloid solution and 4 parts of blood); crystalloid solutions may be of intracellular type (Custadiol) or extracellular type (Plegisol). Specific details are given in Table 1. Modes of application range from antegrade versus retrograde versus antegrade plus retrograde, intermittent versus single shot, cold (with or without additional warm induction) versus warm. Thereby antegrade and retrograde refer to the route of application: antegrade application follows anatomical routes and normal coronary circulation via insertion of a cardioplegia line into the aortic root below the aortic cross-clamp, whereas retrograde perfusion is achieved via direct intubation of the coronary sinus.

All in situ hearts receive some noncoronary collateral flow so that intermittent replenishment of cardioplegia is needed to maintain the primary goals of hypothermia for cardiac myocyte metabolic demand reduction, washout of accumulated metabolites, counteraction of acidosis, and provision of a cardioprotective composition to lower perfusion injury before the next period of planned ischemia is initiated. So, what is better?

### 3.1. Blood versus Crystalloid?

Several experimental studies favor the use of blood cardioplegia over crystalloid solutions when comparing release of cardiac enzymes and metabolic response [10]. To our knowledge, Øvrum et al. from the Oslo Heart Center conducted the largest prospectively randomized single center trial comparing postoperative outcomes of cold blood versus cold crystalloid cardioplegic regimens (1440 CABG patients [11] and 345 aortic valve patients [12]). All patients were gender, age, and perioperative risk matched and no statistical significant differences were seen regarding perioperative and postoperative parameters (Table 3). Even in patients with higher operative risk (female sex, age >70 years, unstable angina, diabetes, emergency operation, ejection fraction <50%, crossclamping time >50 minutes, and EuroSCORE II >5), no statistically significant differences could be demonstrated [11, 12].

A large meta-analysis by Guru et al. [13] from the Toronto University compared 34 trials with a total of 5,044 patients. 2,582 received blood cardioplegia and 2,462 received crystalloid cardioplegia (no differentiation between intracellular and extracellular type crystalloid solutions was made). The authors found no difference between groups regarding perioperative and postoperative myocardial infarction and death (*P* = 0.19 and *P* = 0.44, resp.); however, they did observe a significantly lower incidence of low output syndrome (LOS) immediately upon reperfusion with blood cardioplegia (*P* = 0.006). Various definitions of LOS exist in literature and are discussed in Table 2. Also, CKMB release at 24 h after surgery was considerably lower with blood cardioplegia (*P* = 0.007).

### 3.2. Antegrade versus Retrograde versus Antegrade/Retrograde?

Retrograde cardioplegia is an established method of myocardial protection [14–16]. The rationale behind retrograde application is that distribution of antegrade delivered cardioplegia might be impaired due to ventricular hypertrophy or significant coronary artery stenosis and retrograde application, bypassing plagued vessels, might be of advantage. However, the problem with retrograde perfusion is purely anatomical. Many of our colleagues believe that Thebesian veins play a central role in the distribution of retrograde cardioplegia. Of course this cannot be true. By definition, Thebesian veins constitute venous anastomoses directly “escaping” into the atrial and ventricular cavities bypassing the microvasculature and diminishing nutrient flow. This is reflected in the clinical setting by higher perfusion volumes rather than higher perfusion pressures required to induce cardiac arrest. In studies conducted by Gates et al. from UCLA in Los Angeles on human freshly explanted hearts it became obvious that all regions of the heart can be homogenously perfused in a retrograde fashion [17]. However, when collecting the effluents using colored microspheres 67.2 ± 6.4% of retrograde delivered blood cardioplegia was found to exit Thebesian veins directly into heart cavities, whereas only 29.3 ± 6.3% and 3.5 ± 3.1% escaped from the left and right coronaries, respectively [17]. Although all areas of the ventricles are perfused by retrograde blood cardioplegia, the majority of flow is through the Thebesian system and, thus, is nonnutritive. Further, the nutritive capillary flow that does occur is heavily weighted between ventricles and the right side gets only 10% of the nutrient flow when compared to the left ventricle [17]. Vähäsilta et al. [18] used isolated pig hearts to compare antegrade versus retrograde* crystalloid cardioplegia* in a standardized perfusion model. He assessed for cardiomyocyte apoptosis via terminal transferase mediated ddUTP nick end-labeling (TUNEL) assay and immunohistochemical staining for caspase-3 (Table 3). Cardiomyocyte apoptosis was significantly elevated following reperfusion in both groups, suggesting that myocardial protection in general is suboptimal in antegrade and retrograde cardioplegia. In the right ventricle, however, retrograde cardioplegia was associated with a 3.4-fold higher amount of apoptotic cardiomyocytes as compared with antegrade cardioplegia (0.107% versus 0.032%, *P* < 0.05). A similar difference was also found in the left ventricle, although at a lower level (0.027% versus 0.012%, *P* < 0.05). A subsequent clinical study with 20 patients undergoing aortic valve surgery led by the same authors found that cardiomyocyte apoptosis is significantly increased in the left ventricle after the procedure in the retrograde, but not in the antegrade group (0.00% versus 0.092%, *P* = 0.01 and 0.00% versus 0.023%, *P* = 0.14) [19]. Regarding functional data the amplitude of longitudinal systolic motion of the lateral mitral annulus (an indicator of systolic contractile function) was lower after the operation than before in the retrograde (*P* = 0.03) but not in the antegrade group (*P* = 0.78) [19]. There were no differences in cardiac output, velocities of the E or A waves or E/A ratios in either antegrade or retrograde groups. Altogether, retrograde cardioplegia was associated with left better than right ventricular protection as evident by morphometric and functional analysis, however, this finding did not reflect on patient outcome. Findings of increased right ventricular stress with retrograde cardioplegia alone could be confirmed by Lotto et al. from the Bristol Heart Institute comparing 39 patients undergoing elective aortic valve replacement [20]. They also found that troponin I levels were significantly elevated in both groups again suggesting that myocardial protection in general is suboptimal [20]. However, both authors did not observe statistical differences in the postoperative patient outcome.

There is sufficient evidence, however, that combined antegrade and retrograde cardioplegia is of advantage. Radmehr et al. [21] compared antegrade versus antegrade/retrograde cardioplegia in 87 randomly assigned, age, gender, and perioperative risk matched patients undergoing CABG and found that 35.5% versus 19.0% of patients needed inotropic support while weaning from bypass (*P* = 0.04).

### 3.3. Cold versus Warm?

Fan et al. [22] conducted a meta-analysis of warm versus cold cardioplegia identifying 41 randomized controlled trials with 5,879 patients. In-hospital mortality, length of stay, incident of stroke, and atrial fibrillation (Afib) and use of balloon pumps did not differ between groups. However, warm cardioplegia was associated with significantly better postoperative cardiac index (*P* < 0.00001), lower troponin concentrations on day 0 (*P* = 0.006), and significantly lower peak CKMB concentrations (*P* = 0.002). Mallidi et al. [23] performed a prospective single center cohort study comparing patients receiving cold or tepid/warm cardioplegia during isolated CABG on early and late outcomes and found superior outcomes in the warm cardioplegia arm: perioperative death (1.6 versus 2.5%, *P* = 0.027) and myocardial infarction (2.4 versus 5.4%, *P* < 0.0001).

### 3.4. Terminal Warm Induction (Hot-Shot)?

Intermittent antegrade cold-blood cardioplegia followed by terminal warm-blood cardioplegic reperfusion (hot-shot induction) is reported to reduce myocardial injury in the setting of coronary surgery [24]. Caputo et al. [25] from the Bristol Heart Institute compared 35 patients receiving cold blood cardioplegia with or without terminal warm induction prior to removal of the cross-clamp. Significant metabolic derangement occurs in the ischemic-reperfused hearts of patients with cold blood cardioplegia but not in the hot-shot group (*P* < 0.05) as evidenced by high ADP/ATP ratios and an increase in the alanine-glutamate ratio suggesting the occurrence of anaerobic metabolic activity (Table 3) [25]. Troponin I concentrations were consistently higher in the cold blood group, without reaching statistical significance.

A survey of practice in the UK [26] among cardiac consultants from 2004 found that, of the surgeons performing on-pump CABG, 56% use cold blood cardioplegia, 14% use warm blood cardioplegia, 14% use crystalloid cardioplegia, 21% use retrograde infusion, and 16% do not use any cardioplegia (cross-clamp fibrillation). This impressively highlights that there exists no consensus in the cardiac surgery community regarding the type and route of cardioplegia application.

## 4. Ischemia and Reperfusion on the Blueprint: A Double-Edged Sword?

Since the initial description of the phenomenon by Jennings et al. [27, 28] (see above) some 50 years ago, our understanding of the underlying mechanisms of ischemia reperfusion injury has grown significantly, yet molecular and cellular events underlying IR injury are complex, representing the confluence of divergent biologic pathways [29].

### 4.1. Ischemia-Microvascular Dysfunction

**Figure 2:**
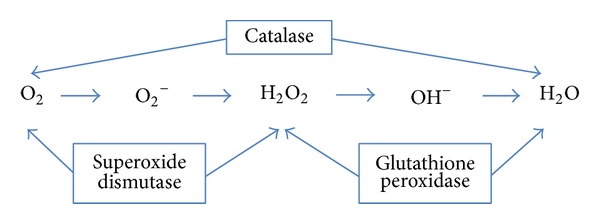
Rapid release of ROS overwhelms the cellular defense mechanisms and leads to toxic cell damage.

The microcirculation represents the major target site of ischemia reperfusion (IR) injury [30–33]. Response to ischemia, initially compensatory and adaptive in nature, progresses to structural changes that become self-perpetuating and pathogenic, when sustained for more than a couple of minutes. Ion influx and cellular swelling impair reperfusion (no-reflow phenomenon) as energetic imbalance characterized by both decreased oxidative phosphorylation and impaired cellular energy production causes tissue acidosis and reactive oxygen species (ROS) formation leading to toxic cell damage [34]. Thereby, ROS generated as the final electron acceptor (O_2_) in the electron chain is insufficient in quantity and O_2_ cannot be further reduced to H_2_O (Figure 2) and the electron transport chain comes to a hold.

Reperfusion in turn, as a growing body of evidence unveils, triggers, an inflammatory response via leukocyte transmigration into tissue with concomitant activation and dysfunction of the vascular endothelial cell barrier and mediates both innate and adaptive immunogenic processes via a multitude of cascades [35, 36].

During ischemia, energy rich phosphates are depleted and the cellular active transmembrane ion transports (Na^+^/K^+^ATPase) are working at a lower pace with intracellular sodium buildup and ionic cellular swelling. High sodium concentrations, in turn, drive increases in intracellular Ca^2+^ via Na^+^/Ca^2+^ exchange [37]. Further Ca^2+^ uptake via sarcolemmal L-type channels and impaired sarcoplasmic clearance via sarcoplasmic endoplasmic reticular calcium ATPase (SERCA) further drives a mechanism of self-perpetuating intracellular Ca^2+^ overload [38–41] eventually leading to ultrastructural changes, activation of apoptotic caspase pathways, autophagy-associated cell death, and necrosis [42].

### 4.2. Reperfusion: The Many Faces of Inflammation

Not only is IR limited to capillary events described above but also the postcapillary venules and their lining endothelial cells set an even bigger stage for a variety of molecular effectors to occur upon reperfusion [29, 35, 36, 43]. The central idea of reperfusion is injury-mediated activation of endothelial cells attracting polymorphonuclear leukocytes (PMNs) via upregulation of receptors and signal molecules on both leukocytes and endothelial cells [44] with subsequent transmigration and tissue invasion through a dysfunctional endothelial cell barrier and impaired cell-cell connections with concomitant increased vascular permeability, edema formation and inflammation [36]. The phenotype of inflammatory response to IR and to that observed during microbial infection share many similarities [42]. For example, ligand binding to pattern recognition molecules like TLRs (toll-like receptors) leads to downstream activation of nuclear factor kappa B (NF*κ*B) and mitogen activated protein kinase (MAPK) pathways resulting in increased transcription of proinflammatory cytokines activating both endothelial cells and leukocytes. Surprisingly, TLRs can either be activated by microbial compounds or cellular debris in the context of IR injury [42, 45–47].

**Figure 3:**
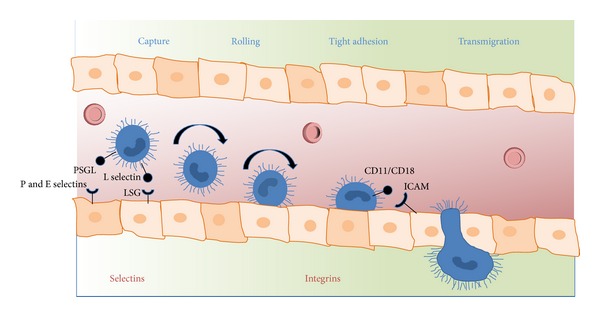
Dance of neutrophils. Leukocyte capture, rolling, adhesion, and transmigration through the endothelial cell barrier. PSGL, P selectin glycoprotein ligand; LSG, L selectin glycoprotein ligand; ICAM, intercellular adhesion molecule. Initial capture of leukocytes is mediated via selectin (endothelial P/E selectin binds PSGL); tight adhesion is mediated via integrins (neutrophil beta-2, leukocyte function associated antigen, CD11/CD18 binds ICAM).

Activation of endothelial cells and PMNs results in upregulation of cellular receptors engaging leukocytes into a dance across endothelial cells (Figure 3) consisting of leucocyte tethering, activation, adhesion, and subsequent transmigration. The ability of PMNs to leave the blood stream and enter surrounding tissues is a critical feature of the immune response [36]. Each of these steps requires either upregulation or activation of distinct sets of adhesion molecules. Thereby, selectins initiate leukocyte attachment along vascular endothelium by mediating leukocyte rolling along activated endothelium (P selectin CD62P binds P selectin glycoprotein ligand 1) whereas integrins such as the neutrophil beta-2 integrin (CD11/CD18) bind endothelial intercellular adhesion molecules (ICAM) and play an important role in the subsequent steps of leukocyte migration into tissues (Figure 3).

Myocardial reperfusion not only saves the majority of the ischemic cells, but paradoxically has a downside called reperfusion injury with further myocardial injury and cardiomyocyte death, in part from microvascular (endothelial) injury and sterile inflammation. Reperfusion damage is a key determinant of postoperative organ functional recovery, morbidity, and mortality in adult and pediatric patients undergoing open-heart surgery. It is, therefore, important to understand the concept of ischemia with subsequent reperfusion as a two-edged sword.

## 5. Experimental Translational Approaches

When preparing our review we found a total of 17.513 patients being compared to the type of cardioplegia they received (Table 3) and there is little evidence that blood or crystalloid, antegrade or retrograde, cold (with or without additional warm induction) or warm have any major impact on clinical outcome. Manipulating the conditions of delivery are, essentially, cosmetic changes. Real changes require innovative steps in the concept of myocardial protection and treatment of ischemia reperfusion injury is the cornerstone of future cardioprotective regimens. The most promising and recent approaches are presented in Table 4.

### 5.1. Is Peptide Treatment a Trojan Horse?

A competent endothelial cell barrier is the center structure integrating various proinflammatory signals and sets course for perfusion injury development. In terms of endothelial cell integrity and fluid extravasation VE cadherin is one of the key molecules integrating signals for opening and tightening of cell junctions [43, 48, 49] and is required for maintaining a restrictive endothelial cell barrier [44]. Barriers are largely formed by endothelial cell-cell contacts built up by VE-cadherin and are under the control of RhoGTPases [43]. VE cadherin has a binding site for the E1 fragment of the fibrin molecule [36]. Interaction of the fibrin *β*-chain and VE cadherin induces capillary tube formation, cell-cell contraction, and upregulation of ICAM-1 expression and induces leukocyte migration [50]. Cell-cell contraction and rupture of cell-cell contacts result in capillary leak. Blockage of the VE cadherin binding site for the fibrin *β*-chain proves promising in reduction of IR injury and leukocyte transmigration [43, 48, 49]. The molecular key for protection is a peptide from the N-terminus of the *β*-chain (B*β*_15–42_) which dissociates the src kinase Fyn from VE-cadherin-containing junctions. Following exposure to B*β*_15–42_, Fyn dissociates from VE-cadherin and associates with p190RhoGAP, a known antagonist of RhoA activation [43]. The activity of RhoGTPases is strictly regulated [51]. Rho GTPases cycle between an active GTP bound and an inactive GDP bound state. Rho GTPases can only interfere with their downstream effectors in the active GTP bound state. The activation of Rho GTPases is mediated by specific guanine nucleotide exchange factors (GEFs), which catalyse the exchange of GDP for GTP. The fibrin derived peptide drug B*β*_15–42_ has been proven to significantly reduce IR injury and leukocyte transmigration in experimental preclinical studies specifically designed with prolonged ischemia times [heart transplantation model in rodents [36, 49, 52], kidney transplantation model in rodents [48], animal models of Dengue shock syndrome [43], burn model in rodents [35], vascular interpolate models [53], and lung transplantation model in rodents (unpublished)] and to significantly decrease the necrotic core zone in acute ST elevation myocardial infarction in a clinical phase II study [54] and may constitute a Trojan horse in future IR injury treatment (Table 4).

### 5.2. Endothelin Receptor Blockers

The central role of the endothelial cell barrier in IR injury is not just confined to the receptors they express or the cytokines they upregulate but also the hormones they secrete [55]. Both circulating endothelin (ET-1) and endothelin receptors are significantly upregulated over ischemic myocardial tissue during IR and are associated with ischemic myocardial contracture [55]. This phenomenon is defined as a rise in resting tension and has attracted quite some attention since the recognition of the “stone heart” as a complication of cardiopulmonary bypass. On isolated rat hearts the ET_A_ selective antagonist PD 155080 (Table 4) reduces peak ischemic contracture (−49%), delays its time to onset (+56%), and improves recovery of reperfusion left ventricular developed pressure (LVDevP +12%), coronary flow (+16%), and diastolic relaxation (+50%) [55].

### 5.3. The Role of Mitochondrial Pores-Cyclosporine Rescue

Cardiac myocytes constitute both highly energy dependent and consuming cellular entities and as such not surprisingly host a high density of mitochondria for electromechanical coupling and force generation. Those cellular batteries can only guarantee function if the innermost mitochondrial membrane is maintained as an impermeable layer for buildup of an electrochemical gradient. Pores in this membrane lead to dissipation of the electric potential across the mitochondrial membrane resulting in “cellular suffocation” in part resembling uncoupling agents and enable mitochondrial-cytosolic escape of reactive oxygen species and induction of apoptosis. As part of this, the mitochondrial permeability transition pore (mPTP) has attracted attention recently as being Ca^2+^ and ROS inducible [56], which directly links mPTP to ischemic injury, and Cyclosporine A sensitive (nonspecific mPTP opening inhibitor) [29, 57]. mPTP seems to be the common effector of a series of upstream signals and Cyclosporine trials on perfusion injury reduction published in the New England Journal of Medicine proved promising [29, 58] (Table 4).

### 5.4. Toll-Like Receptors and TAK-242

Surprisingly, the phenotype of inflammatory response to IR and to that observed during microbial infection is very similar [42]. Ligand binding to pattern recognition molecules like TLRs (toll-like receptors) leads to downstream activation of nuclear factor kappa B (NF*κ*B) and mitogen activated protein kinase (MAPK) pathways resulting in increased transcription of proinflammatory cytokines activating both endothelial cells and leukocytes. TLRs can either be activated by microbial compounds or cellular debris in the context of IR injury [42, 45–47]. One of the most widely investigated pattern recognition molecules is TLR4 usually mediating inflammatory response to gram negative lipopolysaccharide particles (LPS); however, TLR4 activation is significantly enhanced by oxidative stress occurring during IR injury [59]. TAK-242, an inhibitor of TLR4, shows efficacy in reduction of IR injury in large animal trials [60] (Table 4).

### 5.5. The Potential of Endogenous Mechanisms and Prolylhydroxylase

Apart from the lab bench, the heart does have endogenous protective mechanisms to fight ischemia for a limited period of time and those mechanisms might be amenable for future clinical practice as well. One of those endogenous mechanisms can be activated by repeated cycles of brief ischemia. Ischemic preconditioning (IP) is defined as an experimental technique that renders tissues resistant to the deleterious effects of ischemia reperfusion by prior exposure to brief repetitive cycles of vascular occlusion [61]. Cardioprotection by IP involves alteration of the myocardial cell phenotype to become more resistant to subsequent ischemic challenges [61]. During ischemia, energy metabolism switches from fatty acid oxidation to more oxidation-efficient glycolysis, allowing tissues to tolerate ischemic insults for a longer period of time. Thereby, hypoxia inducible factor- (HIF-) 1 acts as a molecular switch [62]. The stability of HIF is actually regulated by the oxygen-sensing prolylhydroxylase (PHD) enzyme. Convincing evidence suggests hypoxia inducible factor- (HIF-) 1 to play a central role in cardioprotection during IP.  HIF-1 resides in the cytosol and translocates into the nucleus for subsequent gene expression of protective pathways upon anoxic insults [61]. When oxygen is abundant, HIF-1 is degraded mediated by Van Hippel Lindau tumor suppressor and PHD [61]. Treatment with pharmacological PHD inhibitors results in increased ischemia tolerance of the kidneys [63] and in cardioprotection similar to that seen with ischemic preconditioning in the heart [61]. To date, PHD inhibitors seem to be well tolerated in humans   [64] suggesting that they could be readily tested in larger clinical trials (Table 4).

Recent experiments suggests that, for activation of this self-protective mechanisms, it is not relevant whether conditioning ischemic cycles precede (preconditioning) or follow (postconditioning) the sustained myocardial ischemia or whether they occur in organs remote from the heart (remote conditioning) [65].

## 6. Conclusion

Taken together, this review shows that cardioprotection has evolved from pure application of potassium rich solution towards a complex field in which different and, in part, adversarial strategies exist: ranging from different routes of administration (antegrade/retrograde) via different preparations (crystalloid/blood) to different modes (warm/tepid/warm, single shot/intermittent). But despite this multitude of options, current clinical regimens might be less than optimal for certain patient collectives and certain clinical scenarios. Current cardioplegic regimens were originally developed in the setting of CABG procedures, with morphologically relatively unchanged hearts. But the field of cardiac surgery is continuously changing while the patient collective undergoing cardiac surgery is becoming more complex.

In an area in which current cardioprotective regimens are limited and no longer adequately meet the demands of a still growing and high-rising cardiothoracic surgery community's expectations, one possibly needs to redefine cardioprotective regimens.

This review highlights both improved understanding and improved management of ischemia reperfusion injury as the central dogma of future cardioprotective approaches and draws attention towards potential new clinical avenues with a considerable translational perspective. Chances are good that we find one of those promising approaches as part of future cardioprotective regimens.

## Figures and Tables

**Table 1:** Composition of crystalloid (intracellular type: Custodiol, HTK, Bretschneider's, and extracellular type: Plegisol, St. Thomas solution) and blood based cardioplegic solutions.

Formulation ingredient	Crystalloid-based cardioplegia		Blood-based cardioplegia		Units
	Intracellular Custodiol, HTK, Bretschneider's	Extracellular Plegisol, St. Thomas solution	Blood cardioplegia induction 4 : 1	Blood cardioplegia maintenance 4 : 1	
Na^+^	15	110	140	140	mmol/L
K^+^	9	16	20	10	mmol/L
Mg^2+^	4	16	13	9	mmol/L
Ca^2+^	0.015	1.2	—	—	mmol/L
Histidine	198	—	—	—	mmol/L
Tryptophan	2	—	—	—	mmol/L
Ketoglutarate	1	—	—	—	mmol/L
Mannitol	30	—	—	—	mmol/L
Glucose	—	—	6	6	mmol/L
Lidocaine	—		260	—	mg/L
pH	7.02–7.20	7.8	7.2	7.4	[H^+^]

**Table 2:** Definitions of low output syndrome (LOS) used in 34 trials. Adapted from [13].

Definitions of LOS	
Requirement of inotropes for >30 minutes or IABP for maintenance of blood pressure >80 mm Hg	
The need for inotropic and/or IABP assistance to maintain the systolic BP at a level >90 mm Hg for at least 30 minutes in the ICU	
Requirement for inotropic agents or IABP for hypotension	
Use of inotropic agents or IABP assistance for hypotension	
CI <2.01 L/min/m^2^ and the need for dopamine (>4 *μ*g/min/kg), dobutamine, or adrenaline administration for >30 minutes or IABP	
CI <2 L/m^2^ despite PAWP ≥15 mm Hg, dopamine in a dose of 3–4.9 *μ*g/kg/min, and, if required, adrenaline in a dose of 0.02–0.10 *μ*g/kg/min	
Unusual need of inotropic (>6 U dopamine) or mechanical (IABP/VAD) support to maintain the normal CO of the patient (normal CO 4–8 L/min)	
CI <2.1 L/min/m^2^	
Systolic BP <90 mm Hg and mixed venous oxygen saturation <60% despite adequate preload and afterload	

**Table 3:** Best evidence papers.

Authors	Title	Year	Intervention	Patients included	Study design	Study endpoints	Reference
Øvrum et al.	A prospective randomized study of 1440 patients undergoing coronary artery bypass grafting	2004	Blood versus crystalloid	1.440∗	Prospective randomized	Operative variables^1^, inotropic support, ICU/hospital stay, arrhythmias, stroke, mortality	[11]
Ovrum et al.	A prospective randomised study of 345 aortic valve patients	2010		345∗	Prospective randomised		[12]
Guru et al.	Is blood superior to crystalloid cardioplegia? A meta-analysis of randomized clinical trials	2006		5.044∗	Meta-analysis	LOS, MI, CKMB at 7 h, 24 h, 48 h	[13]
Vhsilta et al.	Cardiomyocyte apoptosis after antegrade and retrograde cardioplegia during aortic valve surgery	2011	Antegrade versus retrograde	20∗	Prospective randomised	Cardiomyocyte apoptosis (TUNEL assay, caspase 3, BCL-2, and BAX via ventricular biopsies upon reperfusion) ECHO	[19]
Lotto et al.	Myocardial protection with intermittent cold blood during aortic valve operation: antegrade versus retrograde delivery	2003		39∗	Prospective randomised	Biopsies 20 min after cross-clamp removal, adenine nucleotide metabolites, lactate, troponin I	[20]
Radmehr et al.	Does combined antegrade-retrograde cardioplegia have any superiority over antegrade cardioplegia?	2008	Antegrade plus retrograde	87∗	Prospective randomised	Inotropic support morbidity, ICU/hospital stay, mortality	[21]
Fan et al.	Does combined antegrade-retrograde cardioplegia have any superiority over antegrade cardioplegia?	2010	Warm versus cold	5.879∗	Meta-analysis	LOS, inotropic support, MI, stroke, arrhythmias, cardiac index, Troponin, CKMB	[22]
Mallidi et al.	The short-term and long-term effects of cold or tepid cardiopelgia	2003		6.064∗	Prospective cohort	MI, Mortality	[23]
Caputo et al.	Warm blood hyperkalaemic reperfusion (hot shot) prevents myocardial substrate derangement in patients undergoing coronary artery bypass surgery	1998	Cold-cold plus hot-shot	35∗	Prospective randomised	Adenine nucleotide metabolites, alanine-glutamate ratio, lactate, troponin I: 5 min after begin of bypass, 30 min after arrest and 20 min after reperfusion	[25]

LOS, low output syndrome (requiring intotropic and/or intra-aortic balloon pump support); MI, myocardial infarction; CKMB, creatinine kinase MB; BCL-2, B-cell lymphoma 2 (antiapoptotic) and BAX protein (proapoptotic). ^1^Operative variables include amount of cardioplegia used, spontaneous sinus rhythm after declamping, atrioventricular block, fluid excess. ∗7 prospective randomized studies as well as 2 large meta-analyses yielded a total number of **17.513 patients**. The meta-analysis performed by Guru et al. [13] included the initial Ovrum et al. [12] study and those patients were thus subtracted.

**Table 4:** Examples of promising therapeutic approaches targeting ischemia reperfusion injury. Adapted from [42].

Intervention	Target	Potential downside	Stage	Reference
Fibrinogen split product B*β*_15–42_	VE Cadherin	Unclear	Preclinical	[35, 36, 43, 48, 49, 53, 54, 66]
Cingulin derived sequence GRRPGGISGG	RhoAGTPase and VE Cadherin	Unclear	Preclinical	[35]
TAK-242	TLR4	Immunosuppression	Phase II clinical trial	[60, 67]
Cyclosporine	Cyclophilin/mPTP	Immunosuppression	Phase II clinical trial	[58]
PHD inhibitor	Oxygen sensing PHD enzyme, HIF stabilization	Unclear	Phase II clinical trial	[61, 63]
Ischemic preconditioning	Multiple	Unclear	Phase II clinical trial	[61, 68]
Ischemic postconditioning	Multiple	Unclear	Phase II clinical trial	[69]
Remote ischemic conditioning	Multiple	Unclear	Phase II clinical trial	[70]
Endothelin blockers	Endothelin A receptor/Na^+^/H^+^ exchange	Hypotension	Phase II clinical trial	[55, 71]

## References

[1] Chambers DJ, Fallouh HB (2010). Cardioplegia and cardiac surgery: pharmacological arrest and cardioprotection during global ischemia and reperfusion. *Pharmacology & Therapeutics*.

[2] Ghanta RK, Shekar PS, McGurk S, Rosborough DM, Aranki SF (2011). Long-term survival and quality of life justify cardiac surgery in the very elderly patient. *The Annals of Thoracic Surgery*.

[3] Nicolini F, Agostinelli A, Vezzani A (2014). The evolution of cardiovascular surgery in elderly patient: a review of current options and outcomes. *BioMed Research International*.

[4] Hassan A, Newman A, Ko DT (2010). Increasing rates of angioplasty versus bypass surgery in Canada, 1994–2005. *American Heart Journal*.

[5] Ingwall JS (2009). Energy metabolism in heart failure and remodelling. *Cardiovascular Research*.

[6] Massberg S, Messmer K (1998). The nature of ischemia/reperfusion injury. *Transplantation Proceedings*.

[7] Fey K, Follette D, Livesay J (1977). Effects of membrane stabilization on the safety of hypothermic arrest after aortic cross-clamping. *Circulation*.

[8] Goldstein SM, Nelson RL, McConnell DH, Buckberg GD (1977). Effects of conventional hypothermic ischemic arrest and pharmacological arrest on myocardial supply/demand balance during aortic cross clamping. *The Annals of Thoracic Surgery*.

[9] Traverso LW, Ferrari BT, Buckberg GD, Tompkins RK (1977). Elevated postoperative renal clearance of amylase without pancreatitis after cardiopulmonary bypass. *The American Journal of Surgery*.

[10] Barner HB (1991). Blood cardioplegia: a review and comparison with crystalloid cardioplegia. *The Annals of Thoracic Surgery*.

[11] Øvrum E, Tangen G, Tølløfsrud S, Øystese R, Ringdal MAL, Istad R (2004). Cold blood cardioplegia versus cold crystalloid cardioplegia: a prospective randomized study of 1440 patients undergoing coronary artery bypass grafting. *The Journal of Thoracic and Cardiovascular Surgery*.

[12] Øvrum E, Tangen G, Tølløfsrud S, Øystese R, Ringdal M-AL, Istad R (2010). Cold blood versus cold crystalloid cardioplegia: a prospective randomised study of 345 aortic valve patients. *European Journal of Cardio-thoracic Surgery*.

[13] Guru V, Omura J, Alghamdi AA, Weisel R, Fremes SE (2006). Is blood superior to crystalloid cardioplegia? A meta-analysis of randomized clinical trials. *Circulation*.

[14] Anderson WA, Berrizbeitia LD, Ilkowski DA (1995). Normothermic retrograde cardioplegia is effective in patients with left ventricular hypertrophy: a prospective and randomized study. *The Journal of Cardiovascular Surgery*.

[15] Dagenais F, Pelletier LC, Carrier M (1999). Antegrade/retrograde cardioplegia for valve replacement: a prospective study. *The Annals of Thoracic Surgery*.

[16] Menasche P, Tronc F, Nguyen A (1994). Retrograde warm blood cardioplegia preserves hypertrophied myocardium: a clinical study. *Annals of Thoracic Surgery*.

[17] Gates RN, Laks H, Drinkwater DC (1993). Gross and microvascular distribution of retrograde cardioplegia in explanted human hearts. *The Annals of Thoracic Surgery*.

[18] Vähäsilta T, Saraste A, Kytö V (2005). Cardiomyocyte apoptosis after antegrade and retrograde cardioplegia. *Annals of Thoracic Surgery*.

[19] Vähäsilta T, Malmberg M, Saraste A (2011). Cardiomyocyte apoptosis after antegrade and retrograde cardioplegia during aortic valve surgery. *The Annals of Thoracic Surgery*.

[20] Lotto AA, Ascione R, Caputo M (2003). Myocardial protection with intermittent cold blood during aortic valve operation: antegrade versus retrograde delivery. *The Annals of Thoracic Surgery*.

[21] Radmehr H, Soleimani A, Tatari H, Salehi M (2008). Does combined antegrade-retrograde cardioplegia have any superiority over antegrade cardioplegia?. *Heart, Lung & Circulation*.

[22] Fan Y, Zhang A-M, Xiao Y-B, Weng Y-G, Hetzer R (2010). Warm versus cold cardioplegia for heart surgery: a meta-analysis. *European Journal of Cardio-thoracic Surgery*.

[23] Mallidi HR, Sever J, Tamariz M (2003). The short-term and long-term effects of warm or tepid cardioplegia. *The Journal of Thoracic and Cardiovascular Surgery*.

[24] Ascione R, Suleiman SM, Angelini GD (2008). Retrograde hot-shot cardioplegia in patients with left ventricular hypertrophy undergoing aortic valve replacement. *Annals of Thoracic Surgery*.

[25] Caputo M, Dihmis WC, Bryan AJ, Suleiman M-S, Angelini GD (1998). Warm blood hyperkalaemic reperfusion (“hot shot”) prevents myocardial substrate derangement in patients undergoing coronary artery bypass surgery. *European Journal of Cardio-thoracic Surgery*.

[26] Karthik S, Grayson AD, Oo AY, Fabri BM (2004). A survey of current myocardial protection practices during coronary artery bypass grafting. *Annals of the Royal College of Surgeons of England*.

[27] Jennings RB, Sommers HM, Smyth GA, Flack HA, Linn H (1960). Myocardial necrosis induced by temporary occlusion of a coronary artery in the dog. *Archives of Pathology*.

[28] Murry CE, Jennings RB, Reimer KA (1986). Preconditioning with ischemia: a delay of lethal cell injury in ischemic myocardium. *Circulation*.

[29] Turer AT, Hill JA (2010). Pathogenesis of myocardial ischemia-reperfusion injury and rationale for therapy. *The American Journal of Cardiology*.

[30] Motoyama H, Chen F, Ohsumi A (2013). Protective effect of plasmin in marginal donor lungs in an ex vivo lung perfusion model. *The Journal of Heart and Lung Transplantation*.

[31] Westaby S, Kharbanda R, Banning AP (2012). Cardiogenic shock in ACS. Part 1: prediction, presentation and medical therapy. *Nature Reviews Cardiology*.

[32] Kaszaki J, Wolfárd A, Szalay L, Boros M (2006). Pathophysiology of ischemia-reperfusion injury. *Transplantation Proceedings*.

[33] Frangogiannis NG, Smith CW, Entman ML (2002). The inflammatory response in myocardial infarction. *Cardiovascular Research*.

[34] Galasso G, Schiekofer S, D'Anna C (2014). No-reflow phenomenon: pathophysiology, diagnosis, prevention, and treatment. A review of the current literature and future perspectives. *Angiology*.

[35] Goertz O, Lauer H, von der Lohe L (2014). Peptide XIB13 reduces capillary leak in a rodent burn model. *Microvascular Research*.

[36] Petzelbauer P, Zacharowski PA, Miyazaki Y (2005). The fibrin-derived peptide B*β*15-42 protects the myocardium against ischemia-reperfusion injury. *Nature Medicine*.

[37] Tani M, Neely JR (1989). Role of intracellular na+ in ca2+ overload and depressed recovery of ventricular function of reperfused ischemic rat hearts. Possible involvement of h+-na+ and na+-ca2+ exchange. *Circulation Research*.

[38] Bush LR, Romson JL, Ash JL, Lucchesi BR (1982). Effects of diltiazem on extent of ultimate myocardial injury resulting from temporary coronary artery occlusion in dogs. *Journal of Cardiovascular Pharmacology*.

[39] du Toit EF, Opie LH (1992). Modulation of severity of reperfusion stunning in the isolated rat heart by agents altering calcium flux at onset of reperfusion. *Circulation Research*.

[40] Krause S, Hess ML (1984). Characterization of cardiac sarcoplasmic reticulum dysfunction during short-term, normothermic, global ischemia. *Circulation Research*.

[41] Kaplan P, Hendrikx M, Mattheussen M, Mubagwa K, Flameng W (1992). Effect of ischemia and reperfusion on sarcoplasmic reticulum calcium uptake. *Circulation Research*.

[42] Eltzschig HK, Eckle T (2011). Ischemia and reperfusion—from mechanism to translation. *Nature Medicine*.

[43] Gröger M, Pasteiner W, Ignatyev G (2009). Peptide B*β*15-42 preserves endothelial barrier function in shock. *PLoS ONE*.

[44] Dejana E, Vestweber D (2013). The role of VE-cadherin in vascular morphogenesis and permeability control. *Progress in Molecular Biology and Translational Science*.

[45] Chen GY, Nuñez G (2010). Sterile inflammation: sensing and reacting to damage. *Nature Reviews Immunology*.

[46] Iyer SS, Pulskens WP, Sadler JJ (2009). Necrotic cells trigger a sterile inflammatory response through the Nlrp3 inflammasome. *Proceedings of the National Academy of Sciences of the United States of America*.

[47] McDonald B, Pittman K, Menezes GB (2010). Intravascular danger signals guide neutrophils to sites of sterile inflammation. *Science*.

[48] Sörensen I, Rong S, Susnik N (2011). B*β*
_15–42_ attenuates the effect of ischemia—reperfusion injury in renal transplantation. *Journal of the American Society of Nephrology*.

[49] Wiedemann D, Schneeberger S, Friedl P (2010). The fibrin-derived peptide B*β*15-42 significantly attenuates ischemia-reperfusion injury in a cardiac transplant model. *Transplantation*.

[50] Harley SL, Sturge J, Powell JT (2000). Regulation by fibrinogen and its products of intercellular adhesion molecule-1 expression in human saphenous vein endothelial cells. *Arteriosclerosis, Thrombosis, and Vascular Biology*.

[51] Beckers CML, van Hinsbergh VWM, van Nieuw Amerongen GP (2010). Driving Rho GTPase activity in endothelial cells regulates barrier integrity. *Thrombosis and Haemostasis*.

[52] Zacharowski K, Zacharowski PA, Friedl P (2007). The effects of the fibrin-derived peptide B*β*15-42 in acute and chronic rodent models of myocardial ischemia-reperfusion. *Shock*.

[53] Reisinger U, Schwaiger S, Zeller I (2009). Leoligin, the major lignan from Edelweiss, inhibits intimal hyperplasia of venous bypass grafts. *Cardiovascular Research*.

[54] Atar D, Petzelbauer P, Schwitter J (2009). Effect of intravenous FX06 as an adjunct to primary percutaneous coronary intervention for acute st-segment elevation myocardial infarction: results of the F.I.R.E. (efficacy of FX06 in the prevention of myocardial reperfusion injury) trial. *Journal of the American College of Cardiology*.

[55] Brunner F, Opie LH (1998). Role of endothelin-a receptors in ischemic contracture and reperfusion injury. *Circulation*.

[56] Orrenius S, Zhivotovsky B, Nicotera P (2003). Regulation of cell death: the calcium-apoptosis link. *Nature Reviews Molecular Cell Biology*.

[57] Fancelli D, Abate A, Amici R (2014). Cinnamic anilides as new mitochondrial permeability transition pore inhibitors endowed with ischemia-reperfusion injury protective effect in vivo. *Journal of Medicinal Chemistry*.

[58] Piot C, Croisille P, Staat P (2008). Effect of cyclosporine on reperfusion injury in acute myocardial infarction. *The New England Journal of Medicine*.

[59] Powers KA, Szászi K, Khadaroo RG (2006). Oxidative stress generated by hemorrhagic shock recruits toll-like receptor 4 to the plasma membrane in macrophages. *The Journal of Experimental Medicine*.

[60] Fenhammar J, Rundgren M, Forestier J, Kalman S, Eriksson S, Frithiof R (2011). Toll-like receptor 4 inhibitor TAK-242 attenuates acute kidney injury in endotoxemic sheep. *Anesthesiology*.

[61] Eckle T, Kohler D, Lehmann R, Kasmi KCE, Eltzschig HK (2008). Hypoxia-inducible factor-1 is central to cardioprotection a new paradigm for ischemic preconditioning. *Circulation*.

[62] Eltzschig HK, Carmeliet P (2011). Hypoxia and inflammation. *The New England Journal of Medicine*.

[63] Hill P, Shukla D, Tran MGB (2008). Inhibition of hypoxia inducible factor hydroxylases protects against renal ischemia-reperfusion injury. *Journal of the American Society of Nephrology*.

[64] Bernhardt WM, Wiesener MS, Scigalla P (2010). Inhibition of prolyl hydroxylases increases erythropoietin production in ESRD. *Journal of the American Society of Nephrology*.

[65] Heusch G, Boengler K, Schulz R (2008). Cardioprotection: nitric oxide, protein kinases, and mitochondria. *Circulation*.

[66] Roesner JP, Petzelbauer P, Koch A (2009). A double blind, single centre, sub-chronic reperfusion trial evaluating FX06 following haemorrhagic shock in pigs. *Resuscitation*.

[67] Rice TW, Wheeler AP, Bernard GR (2010). A randomized, double-blind, placebo-controlled trial of TAK-242 for the treatment of severe sepsis. *Critical Care Medicine*.

[68] Petrowsky H, McCormack L, Trujillo M, Selzner M, Jochum W, Clavien PA (2006). A prospective, randomized, controlled trial comparing intermittent portal triad clamping versus ischemic preconditioning with continuous clamping for major liver resection. *Annals of Surgery*.

[69] Staat P, Rioufol G, Piot C (2005). Postconditioning the human heart. *Circulation*.

[70] Bøtker HE, Kharbanda R, Schmidt MR (2010). Remote ischaemic conditioning before hospital admission, as a complement to angioplasty, and effect on myocardial salvage in patients with acute myocardial infarction: a randomised trial. *The Lancet*.

[71] Ryu SM, Kim HJ, Cho KR, Jo W-M (2009). Myocardial protective effect of tezosentan, an endothelin receptor antagonist, for ischemia-reperfusion injury in experimental heart failure models. *Journal of Korean Medical Science*.

